# Disability free life expectancy: a simulation study of scenarios to extend healthy years and reduce inequalities

**DOI:** 10.1186/s12916-026-04934-5

**Published:** 2026-05-21

**Authors:** David R Sinclair, Laurie E Davies, Barbara Hanratty, Chris Todd, Fiona E Matthews, Andrew Kingston

**Affiliations:** 1 National Institute for Health and Care Research (NIHR) Policy Research Unit in Healthy Ageing, Newcastle University, Newcastle upon Tyne, UK; 2 National Institute for Health and Care Research (NIHR) Policy Research Unit in Healthy Ageing, The University of Manchester, Manchester, UK; 3 Institute for Clinical and Applied Health Research, University of Hull, Hull, UK

**Keywords:** DFLE, Healthy Ageing, Health Expectancy, Deprivation, Multistate model, Inequalities, Interventions, Policy

## Abstract

**Background:**

The gap in Disability-Free Life Expectancy between affluent and deprived areas of England is stark, at over 15 years. Successive governments have recognised the need to narrow this and extend the years of life spent without disability, but there is little evidence outlining how large an intervention must be to achieve meaningful gains. This study examines intervention scenarios to (i) extend Disability-Free Life Expectancy and (ii) reduce socioeconomic inequalities in Disability-Free Life Expectancy, among older people in England.

**Methods:**

We applied multistate modelling to longitudinal data on 16 899 individuals, aged 50 + in England, incorporating disability data from three cohort studies: the English Longitudinal Study of Ageing, the Cognitive Function and Ageing Study II, and the Newcastle 85 + Study. Simulations assessed how reducing the risk of disability associated with age and area-based socioeconomic deprivation could extend Disability-Free Life Expectancy. In these simulations, deprivation-targeted interventions reduced the excess disability risk and differential recovery observed in people living in the 20% most deprived areas. Age-targeted interventions reduced the age-related increase in disability risk and the corresponding decline in recovery.

**Results:**

Interventions targeted solely at the most deprived quintile yielded modest Disability-Free Life Expectancy gains (up to 2.8 years for women and 2.3 years for men, in deprived areas only). Interventions targeting age-related disability risk alone were associated with increases in Disability-Free Life Expectancy of 6.3 to 8.7 years under a 40% reduction in age-related disability risk, but exacerbated the gap between the most and least deprived populations. Interventions addressing both age- and deprivation-associated risks demonstrated the greatest potential. A 30% decrease in the age-based probability of disability, and commensurate increases in recovery from disability, alongside removal of deprivation-associated inequalities, increased Disability-Free Life Expectancy by 4.8 to 8.6 years for men and women aged 50, with women living in deprived areas benefiting most.

**Conclusions:**

Extending Disability-Free Life Expectancy while reducing socioeconomic inequality is difficult, but possible by tackling both age- and deprivation-related risks. Taken on their own, age-based interventions risk increasing inequalities, as they disproportionately benefit people living in less deprived areas.

**Supplementary Information:**

The online version contains supplementary material available at 10.1186/s12916-026-04934-5.

## Background

Decreasing the time spent in preventable ill health is a challenge for many countries with ageing populations. Time spent in poor health towards the end of life has implications for the sustainability of health and care services, affordable pension provision, and quality of life [1].

Ill health is a broad concept encompassing disease, multimorbidity and functional limitation. In this study, we focus specifically on disability, defined as difficulty performing activities necessary for independent living, such as self-care (e.g. dressing, toileting) or household tasks (e.g. cooking, shopping) [2–4]. Disability reflects functional capacity rather than the presence of diagnosed disease. Disability-Free Life Expectancy (DFLE) measures the expected number of years a person will live without such functional limitations.

Interventions are needed to narrow health inequalities between the rich and the poor, which are large and have widened over time [5]. For example, people born in the most affluent parts of England will live disability-free for an additional 15 years, on average, than those in the least affluent parts [6]. Recent research also suggests that men in the most disadvantaged areas of England reach the age at which half of their remaining life is spent with disability 11 years earlier than their least disadvantaged counterparts, and for women this difference is even greater, at 12 years [7]. Risk factors for disability, such as smoking, obesity and multimorbidity, are, for example, more common in disadvantaged areas [8–10]. These patterns are shaped not only by individual behaviours, but also by inequalities in the wider social determinants of health, including income, education, employment, housing, neighbourhood environments, and access to health and social care [11, 12].

Disability, which rises with age, is associated with a range of adverse outcomes, including poor quality of life, greater health and social care needs, and premature mortality [13–19]. Older people generally prefer to remain living at home as they age [20]. However, disability can make this difficult, and declining numbers of unpaid carers may further limit support in the home [21]. Extending the number of years people can expect to live free from disability is therefore an important health outcome.

However, to our knowledge, studies have yet to simulate the scale of interventions needed to increase DFLE or narrow its socioeconomic inequalities. This is despite the pledge of the UK government (in 2024) to halve the gap in healthy life expectancy between rich and poor areas, adapting the previous administration’s goal (2018–2023) of extending ‘healthy, independent’ years by 5 years, while simultaneously narrowing the socioeconomic gap, thereby requiring gains in population health that do not widen existing inequalities [22, 23].

Healthy life expectancy is closely aligned with DFLE, as both combine mortality with a measure of years lived in good health [24–26]. They are both measures of health expectancy, although they differ conceptually and methodologically. We focus on DFLE, which captures functional independence. In contrast, healthy life expectancy is an estimate of time spent in self-perceived ‘very good’ or ‘good’ health [24]. DFLE reflects the ability to live independently, and is closely aligned with the concept of ‘healthy, independent years’. By targeting measurable outcomes of functional health, DFLE provides a robust and comparable outcome for assessing the impact of interventions over time and across populations, making it a practical metric for evaluating public health outcomes. Moreover, DFLE informs health care needs, economic impact and societal benefits, and is comparable with other studies [27–29].

This study estimates the scale of change in disability incidence and recovery required to achieve policy-relevant improvements in DFLE. We model a suite of scenarios that alter the rate of disability incidence and recovery in order to find the point at which DFLE is increased by five years, and the gap in DFLE is narrowed between rich and poor areas. This study can inform the development of policies and interventions and provide information on the required efficacy and scale of interventions.

## Methods

### Study population

Longitudinal data on participants’ age, gender, socioeconomic status and disability were sourced from three population-based studies of older people’s health in England: the English Longitudinal Study of Ageing (ELSA) (waves 1–6, 2002–2013) [30, 31], the Cognitive Function and Ageing Study II (CFAS II, 2008–2013) [32, 33], and the Newcastle 85 + study (2006–2016) [34–36] (further details are included in Additional file 1: S1). The inclusion of the Newcastle 85 + Study was primarily to enhance coverage at the oldest ages, as nationally representative cohort studies such as ELSA include relatively small numbers of participants aged 85 years and over, particularly in institutional settings and more deprived areas.

We limited the analysis to participants whose disability or vital status was observed at two or more waves, which is required for longitudinal multistate modelling. Individuals with no recorded disability status at any wave were excluded. Participants without a recorded deprivation quintile were also excluded. This resulted in the inclusion of 10 273 of 11 391 ELSA participants (90.2%), 6 341 of 7 762 CFAS II participants (81.7%), and 838 of 849 Newcastle 85 + Study participants (98.7%).

ELSA and CFAS II use complex sampling designs and provide cohort-specific survey weights to account for differential probabilities of selection and non-response; these weights were applied in the multistate models. The Newcastle 85 + Study was conducted as a census of all individuals born in 1921 within Newcastle and North Tyneside and therefore did not involve sampling probabilities or provide survey weights.

The multistate models estimate individual-level transition intensities conditional on age, gender and deprivation. The pooled sample should therefore be interpreted as representing older adults observed within the most deprived areas and less deprived areas, rather than a single nationally weighted synthetic population.

### Variable definitions

Disability was categorised into three self-reported states: (1) independent, (2) difficulty with mobility or at least one instrumental activity of daily living (IADL) and (3) difficulty with at least one activity of daily living (ADL). ADLs are basic tasks a person needs to perform to live independently and maintain personal care (such as bathing, dressing and eating) and IADLs are more complex skills needed for independent living (such as managing money, cooking and shopping) [2–4]. We separated limitations with mobility/IADLs from ADLs to represent the complex dynamics of ability loss among older people; requiring help for ADLs indicates the most severe and resource-intensive stage of disability [37]. The ADL, IADL and mobility questions asked in ELSA, CFAS II and the Newcastle 85 + study are detailed in Additional file 1: S2. DFLE is the expected number of years lived without disability, that is, without limitations in ADLs, IADLs or mobility, and therefore in an independent state [24].

We used the English Index of Multiple Deprivation (IMD) as a measure of area deprivation [38]. The IMD is the official government metric in England, ranking small geographic areas (1 000–3 000 people) based on seven area-based domains: income, employment, education, health, crime, barriers to housing and services, and the living environment. We use IMD to identify the ‘rich’ and ‘poor’ areas mentioned in government policy. We categorised the most deprived quintile as ‘High deprivation’ and the remaining quintiles as ‘Others’, as life expectancy inequalities in England are steepest at the lower tail of the deprivation distribution [39, 40]. This categorisation simplified the multi-state model, facilitating convergence on the optimal model fit. Deprivation category was fixed at baseline for each participant.

Age (as a continuous variable measured in months) and gender (binary: male/female) were also used as covariates in the models.

### Scenarios

**Fig. 1:**
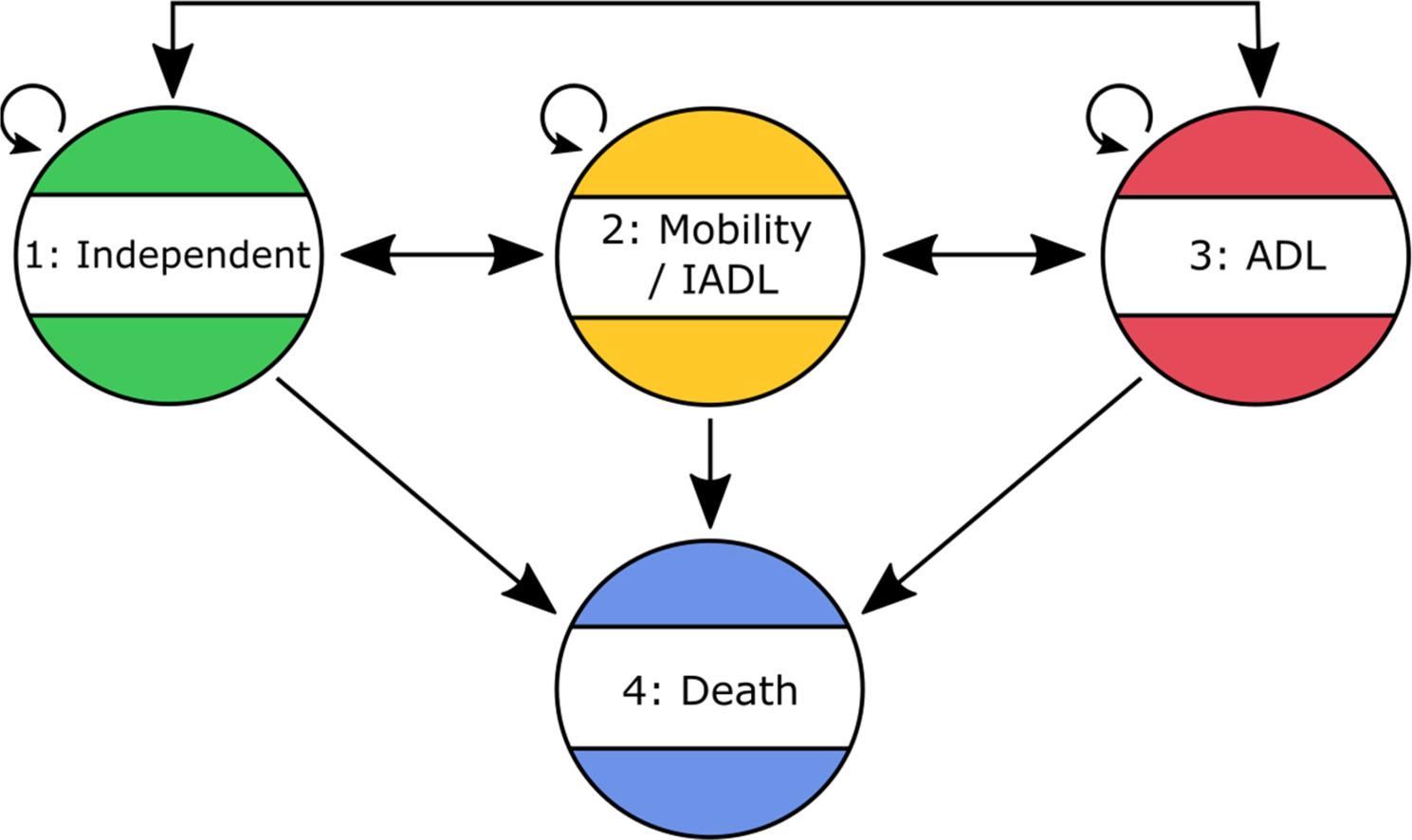
State transition diagram for multi-state model. A person can transition in both directions between the three disability-related states (Independent, Mobility/IADL, ADL) and unidirectionally to the Death state. For the disability states, moving right in the diagram represents transitions to more severe states, while moving left represents recovering to a less disabled state

We created a four-state model to evaluate DFLEs (Fig. 1). Four disability states were used, categorising increasing levels of disability: (1) independent (2), requiring care for mobility/IADLs (3), requiring care for ADLs and (4) death. We modelled bidirectional transitions between the three living states and unidirectional transitions from each living state to death.

We modelled a range of scenarios exploring how changes to age- and deprivation-associated transition probabilities might influence DFLE. Four illustrative scenarios are presented in the main manuscript (Table 1), with further details in Additional file 1: S3 Additional scenarios are reported in Additional file 1: S4-S6. The scenarios presented in the main text were selected to represent contrasting strategic approaches to improving DFLE, rather than to represent specific real-world interventions.

**Table 1:** Four scenarios to extend disability free life expectancies

Intervention Scenario	Modification to Disability Transition Probabilities	Modification to Recovery Transition Probabilities	Deprivation Adjustment	Age-Based Adjustment
**Scenario 1: Deprivation-Based Reduction**	Reduction in transition probability from Independent → Mobility/IADL by 10%	No change	Applied to most deprived quintile only	No adjustments
**Scenario 2: Deprivation Equalisation**	Equalisation of disability rates: people living in the high deprivation quintile move to more disabled states at the same rate as other quintiles	Equalisation of recovery rates: people living in the high deprivation quintile recover to less disabled states at same rate as other quintiles	Eliminates deprivation-associated risk	No adjustments
**Scenario 3: Age-Based Reduction (40%)**	40% reduction in age-related transition probability of moves to more disabled states disability incidence	40% increase in age-related recovery to less disabled states	No changes	Age-based modifications to all ages
**Scenario 4: Combined Age + Deprivation (30%)**	30% reduction in age-related transition probability of moves to more disabled states disability incidence	30% increase in age-related recovery to less disabled states	Eliminates deprivation-associated risk	Both deprivation- and age-based adjustments applied

Summary of four intervention scenarios modelled to extend disability-free life expectancy. Scenarios vary in how they alter age-related and deprivation-related transition probabilities for disability incidence and recovery, either by reducing the risk of progression to more disabled states, increasing recovery to less disabled states, or both

Scenario 1 reduced the age-associated risk of transition from independence to mobility/IADL limitation by 10%, representing a primary prevention strategy. Scenario 2 removed deprivation-associated differences in transition probabilities between disability states (excluding mortality transitions), representing elimination of area-level inequality in disability dynamics. Scenario 3 altered age-associated risks for disability transitions (excluding death) by 40%, decreasing transitions to more disabled states and increasing transitions to less disabled states proportionately. Scenario 4 combined removal of deprivation-associated differences with a 30% modification of age-associated transition risks (excluding death), decreasing the risk of disability transitions and increasing the chance of recovery transitions.

The percentage changes applied in each scenario should be interpreted as illustrative effect-size benchmarks, allowing comparison of how different strategic emphases (age-focused, deprivation-focused, or combined) influence DFLE and inequalities. We did not modify the probability of direct transitions between Independence and ADL limitations, as these events indicate more severe, sudden outcomes from hospitalisation events, rather than more gradual changes in disability which are the focus of this analysis. Further details of the scenario modelling are outlined in Additional file 1: S7.

As individuals age, their probability of transitioning to more severe disability states increases, while their chances of recovery to less disabled states decline. Age-associated scenarios slow the rate at which these transition probabilities change with each year of life. Similarly, individuals living in more deprived areas face a higher risk of progressing to more severe disability states and a lower chance of recovery. Deprivation-associated scenarios aim to reduce these disparities by adjusting transition probabilities for individuals in the most deprived area quintile to more closely resemble those of individuals in less deprived areas.

### Estimation of Disability-Free Life Expectancy (DFLE)

We examined how DFLE changed upon modifying the probability of transitions between disability states in our multi-state models (see Table 1). DFLEs were calculated using Interpolated Markov Chain (IMaCh) software (version 0.99r46) [41].

IMaCh calculates the transition probabilities between disability states using a discrete-time Markov Chain multistate model. Transition probabilities between the states are calculated using multinomial logistic models and use 1-month time steps. In our analysis, transition probabilities were estimated by pooling data from the three cohorts spanning 2002–2016. These probabilities vary based on participant characteristics, which are age, gender and deprivation in our models. IMaCh uses all available observation intervals and accommodates intermittent missing waves or loss to follow-up by estimating transitions between successive observed states, allowing individuals with incomplete follow-up to contribute to the estimation of transition probabilities. The model assumes that these estimated age-, gender- and deprivation-specific transition probabilities are stable over time. Maximum likelihood estimation is used to determine the optimal set of transition probabilities for a given longitudinal data set. Once the transition probabilities are obtained, the expected time spent in each state, can be calculated using a multi-state life table approach, allowing estimation of gender- and deprivation-specific DFLE at age 50 [41].

To validate our life expectancy model, we compared baseline estimates of total life expectancy (i.e., without intervention scenarios) with projections provided by the UK Office for National Statistics [42].

### Target equivalence

Previously stated UK government targets comprise narrowing the gap in healthy life expectancy between rich and poor areas and increasing healthy, independent years by at least five years *at birth* [22, 23]. Our data focuses on adults aged 50 and older, meaning that any target related to life expectancy at birth must be recalibrated for this demographic. People who reach age 50 have already surpassed early-life health risks, meaning that their total DFLE is higher than that of a newborn. This adjustment is even more accentuated for those from deprived backgrounds, as they are the most likely to die before age 50 [43, 44].

To determine target equivalence at age 50, we calculated the proportional increase in DFLE at birth required to achieve a five-year gain within each deprivation group and applied this same proportional increase to DFLE at age 50 (separately by gender and deprivation group), thereby ensuring comparability in relative improvement across population groups. Target equivalence would mean that men and women in the most deprived quintile need an increase of 5.9 and 6.1 years, respectively, to match a five-year increase at birth. In the remaining four quintiles, DFLE needs to increase by 5.5 and 5.6 years for men and women, respectively. This calculation is expanded in Additional file 1: S8.

## Results

### Participant characteristics

The characteristics of the participants in the harmonised data set are shown in Table 2 (with further details in Additional file 1: S9). There were more women (*n* = 9 193) than.

men (*n* = 7 706). 15.4% of women and 14.8% of men lived in the most deprived area quintile, similar to the national proportion of over 50s living in the most deprived area quintile, 16.0% and 16.2% for men and women, respectively [45]. The counts of participants’ transitions between disability states are shown in Additional file 1: S10.

**Table 2:** Counts and percentage of participants

Characteristic	Men	(%)	Women	(%)
	7706	(-)	9193	(-)
Age				
50–54	808	(10.5)	1006	(10.9)
55–59	894	(11.6)	1027	(11.2)
60–64	715	(9.3)	793	(8.6)
65–69	1551	(20.1)	1629	(17.7)
70–74	1311	(17.0)	1430	(15.6)
75–79	1019	(13.2)	1193	(13.0)
80–84	732	(9.5)	1028	(11.2)
≥ 85	676	(8.8)	1087	(11.8)
Deprivation quintile				
1 (Most deprived)	1140	(14.8)	1419	(15.4)
2	1393	(18.1)	1710	(18.6)
3	1558	(20.2)	1885	(20.5)
4	1786	(23.2)	2087	(22.7)
5 (Least deprived)	1829	(23.7)	2092	(22.8)

Counts and percentages of participants in the harmonised dataset by age group, gender and deprivation quintile. Counts represent the first recorded observation for each participant. Additional file 1: S9 reports corresponding counts for the three contributing studies (ELSA, CFAS II and the Newcastle 85 + Study)

**Fig. 2:**
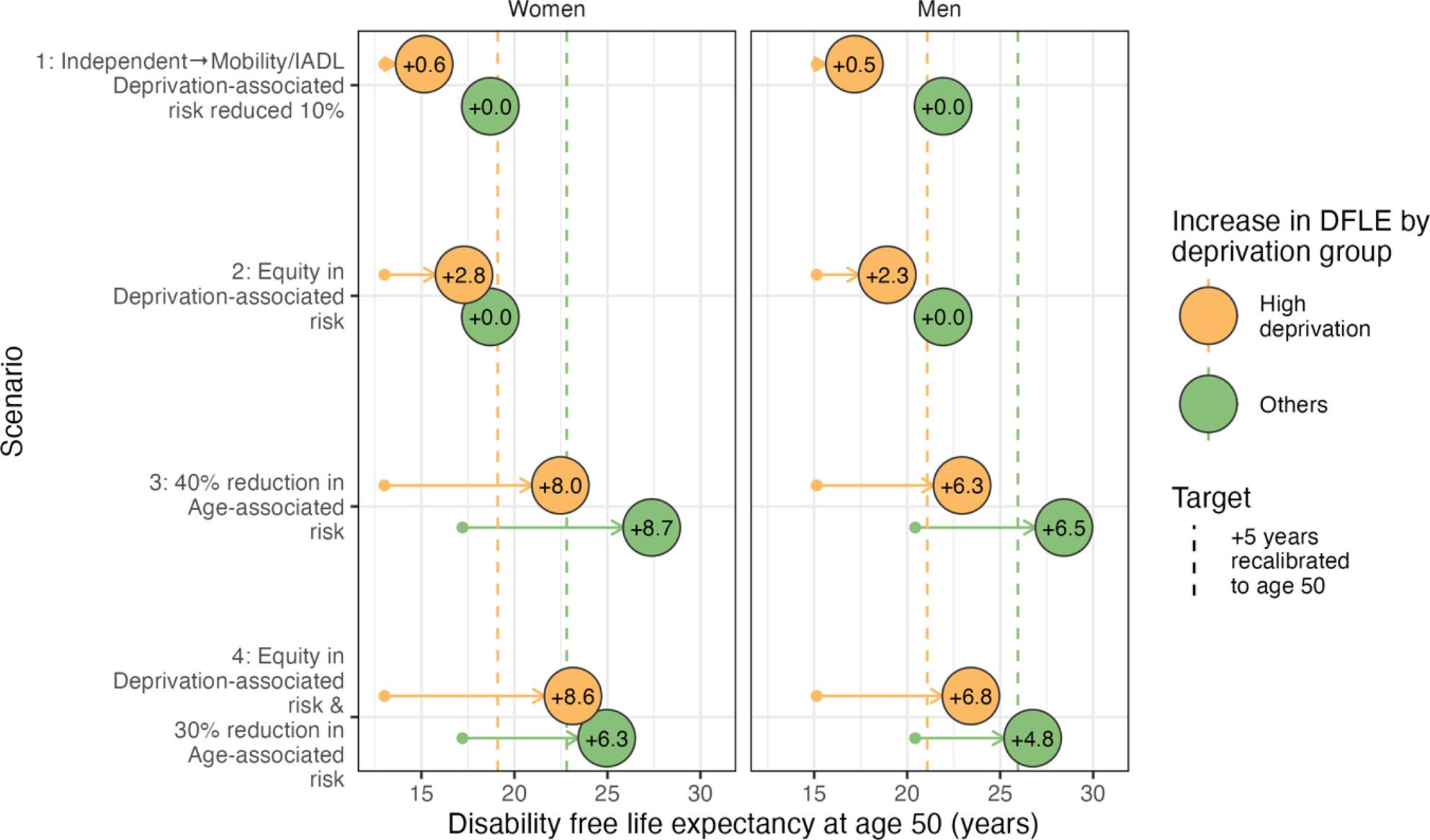
Changes in Disability Free Life Expectancy (DFLE) for four intervention scenarios. Estimated gains in disability-free life expectancy under four illustrative scenarios (see Table 1 for full specification). Dashed lines show an increase of five years’ gain in DFLE at birth, recalibrated for gains from age 50 for each gender and deprivation group

The increases in DFLE from four intervention scenarios are shown in Fig. 2. Tabulated results, including confidence intervals, are presented in Additional file 1: S3. All changes in DFLE are calculated for people aged 50, the youngest age in our dataset.

### Alterations to the deprivation-associated risk of disability

Intervention scenarios which only reduce deprivation-associated risk are insufficient to increase DFLE by five years from birth within deprivation groups (Fig. 2). Reducing the probability of the Independent to Mobility/IADL transition by 10% (Intervention Scenario 1) increases DFLE by only 0.5–0.6 years for only the people living in the most deprived areas. Even an intervention scenario which removed all deprivation-based inequity in the risk of becoming more disabled and the chance of recovering from disability (Intervention Scenario 2) would only extend DFLE by 2.8 years for women living in the most deprived areas and 2.3 years for men.

A range of deprivation-targeted scenarios, including modifications to mortality transitions, are presented in Additional file 1: S4. Among the deprivation-targeted scenarios, equalising recovery from IADL limitation to independence produced the largest gains in DFLE for the most deprived quintile, followed by equalisation of the transition from independence to IADL limitation.

### Alterations to the age-associated risk of disability

An intervention scenario which reduced the age-associated probability of increased disability by 40%, and increased the age-associated probability of recovering to a less disabled state by 40% (Intervention Scenario 3 in Table 1; Fig. 2) would extend DFLE for women and men in the most deprived areas by 8.0 and 6.3 years, respectively. However, it would also extend DFLE for women and men who live in less deprived areas to a greater degree, 8.7 and 6.5 years respectively, therefore exacerbating inequalities.

Further analyses relating to age-associated transition probabilities, including transitions to mortality, are shown in Additional file 1: S5. Within these age-associated scenarios, reducing the transition from independence to IADL limitation produced the largest gains in DFLE.

### Alterations to both the deprivation-associated and age-associated risks of disability

A gain of five years in DFLE while narrowing inequalities can be achieved by targeting both age- and deprivation-associated risks. Changing age-associated transition probabilities by 30% (30% decreased probability of greater disability and 30% increased probability of reduced disability), while also removing deprivation-associated risks (Intervention Scenario 4 ), increased DFLE for women and men living in the most deprived areas by 8.6 and 6.8 years, respectively, and for those in more affluent areas to a lesser degree (6.3 and 4.8 years, respectively). These findings indicate that combined modifications to age- and deprivation-associated transition risks would produce substantial gains in DFLE within both deprivation groups, with larger absolute improvements among those living in the most deprived areas, thereby contributing to a narrowing of inequalities. Although the increase among men living in less deprived areas (4.8 years) falls marginally below the target gain (5.5 years at age 50), it is of a similar magnitude to it. Further analyses relating to both deprivation and age-associated transition probabilities are shown in Additional file 1: S6.

### Total life expectancies and baseline model

To check the validity of our model, we compared the total life expectancy forecast by our baseline model (i.e. without interventions) to those forecast by the UK’s Office for National Statistics (Additional file 1: S11). The standard error of the estimate, based on ages 50–100, between the male estimates is 0.24 years and between the female estimates is 0.67 years.

The baseline (no intervention) model produced transition parameters consistent with established patterns of ageing, gender and disability (Additional file 1: Table S12). Increasing age was associated with higher risks of disability progression and death and lower probabilities of recovery. Women experienced higher probabilities of gradual transitions into disability but lower probabilities of death or direct transitions from independence to ADL disability than men. Living in the most deprived areas increased the likelihood of transitions to more severe disability states. These parameters define the counterfactual trajectory of DFLE onto which intervention scenarios were applied.

## Discussion

### Principal findings

Within the scenarios examined, increasing disability-free life expectancy (in excess of five years), whilst narrowing the gap between those most socioeconomically disadvantaged and everybody else, was only achieved by targeting both age-associated risks of disability and deprivation-associated risks. Of the scenarios examined here three criteria need to be met: First, the age-associated risk of disability must become at least 30% lower. Second, the rate of recovery from more to less disabled states must also increase by at least 30%. Third, the risk of disability for people living in the most deprived areas must decrease, such that it is closer to the risk faced by people living in other areas.

Age-based interventions alone can increase DFLE by five years. However, age-based interventions in isolation expanded the gap in DFLE between people living in the most deprived areas and those living elsewhere.

Interventions that only reduce the gap in DFLE between people living in the most deprived areas and the rest of the population were not sufficient to increase DFLE by five years at birth for people living in the most deprived area quintile. Even fully eliminating these deprivation-related inequalities only increased DFLE by 2.8 years for women and 2.3 years for men - less than half the required gain. Moreover, since this improvement applied only to those in the most deprived areas, there is no increase for people living in less deprived areas.

A DFLE gap remains even after equalising deprivation-associated disability transition probabilities. This reflects two factors. First, individuals in the most deprived quintile are more likely to enter age 50 in a disabled state, meaning that inequalities accumulated earlier in the life course are not removed by altering post-50 transition dynamics. Second, the highlighted models do not modify deprivation differences in mortality risk in the four highlighted scenarios. As DFLE depends on both disability transitions and survival, persistent mortality inequalities continue to limit gains in DFLE among deprived groups. Together, these mechanisms indicate that a substantial portion of the deprivation gradient in DFLE is established prior to age 50 and through differential survival.

This indicates that a portion of the gap is effectively ‘locked in’ through earlier life-course processes and differential mortality, rather than being fully modifiable through later-life disability transitions alone. The model therefore identifies a structural constraint within the 50 + population: interventions confined to this age range, even if highly effective, may not fully eliminate socioeconomic inequalities in DFLE without complementary action earlier in the life course and on mortality disparities.

Of the four intervention scenarios tested, only Scenario 4 (Combined Deprivation and Age – 30%) achieved or approached the five-year (DFLE gain at birth) target within deprivation groups, while narrowing inequalities. In Scenario 4, DFLE for men in less deprived areas increased by 4.8 years, marginally less than the target of 5.5 years (DFLE gain at age 50). However, since these men already have the longest DFLE, they may be considered a lower priority compared to other population groups for DFLE improvement efforts.

The novelty of these findings lies not in demonstrating that reduced disability increases DFLE, but in quantifying the magnitude of change required and showing that strategies focused solely on age-related improvements risk widening inequalities.

### Implications for policy

Public health interventions can be divided into those that address population-level risk and those that target high-risk populations or areas. Our work suggests that both are needed to increase DFLE and narrow the gap between rich and poor areas. This analysis suggests that a multi-faceted approach is needed, as no single area of intervention on disability transition or high-risk groups will suffice.

Age-based interventions risk widening inequalities in DFLE. Individuals of the same age but from different deprivation groups have markedly different baseline risks of disability and mortality, and age-based strategies do little to address the cumulative disadvantages underlying these disparities. Moreover, engagement with interventions is often socially patterned, with lower uptake in more deprived populations, potentially limiting their effectiveness. Taken together, these mechanisms may entrench existing health inequalities unless deprivation is explicitly accounted for in intervention design and delivery. Notably, our results suggest that interventions improving recovery from IADL/Mobility limitation to independence among individuals living in deprived areas could be especially effective in closing socioeconomic gaps in DFLE. This highlights the potential importance of ensuring equitable access to rehabilitation, post-acute care, and functional support services in deprived populations. While prevention remains essential, policies that enhance recovery and functional restoration may be particularly influential in narrowing socioeconomic gaps in later-life health. In the age-based scenarios, preventing or delaying the transition from independence to IADL limitation had the greatest effect on DFLE, suggesting that policies aimed at delaying or preventing this specific transition may be particularly effective in increasing DFLE.

This study does not estimate the feasibility, cost or specific mechanisms through which such changes might be achieved; rather, it provides quantitative benchmarks against which future intervention effectiveness can be assessed. Despite not evaluating specific interventions, the modelled reductions in disability transitions correspond to recognisable areas of policy and practice. Reductions in the transition from independence to IADL limitation could plausibly arise from age-based interventions targeting modifiable risk factors for functional decline, including physical inactivity, obesity and cardiometabolic disease. Improvements in recovery from IADL limitation are associated with expanded access to rehabilitation, reablement and post-acute care [46–48].

These same strategies could be delivered using a deprivation-focused approach if targeted proportionately towards more disadvantaged communities, where multiple long-term conditions and functional limitation occur earlier and recovery may be constrained by fewer resources [49]. In addition, interventions addressing material conditions in later life, including housing quality, income security and employment conditions, may influence both disability onset and recovery [11].

While various approaches are likely to be effective in preventing the onset of disability and supporting recovery, the central concern is that ageing populations will place increasing pressure on health and social care systems if improvements in DFLE do not keep pace [50]. Improvements in DFLE may also have wider economic and societal implications. In the UK, health-related economic inactivity has risen in recent years, particularly among people aged 50–64, with long-term sickness, disability and caring responsibilities leading reasons for labour market exit [51]. Extending DFLE could support longer labour market participation, particularly as State Pension age rises [52].

Our use of data from cohorts aged 50 + leads us to suggest it will be difficult to increase disability-free life expectancy by five years by targeting the age 50 + population. While our analysis does not identify the optimal point for intervention in the life course, it is important to acknowledge that health inequalities are cumulative, operating across the lifespan. Addressing inequalities in childhood and midlife may be essential to reducing the risk of disability in later life, aligning with cumulative disadvantage theory [53]. Early-life interventions, such as obesity prevention, could play a crucial role in preventing individuals from reaching older age with an elevated risk of disability [8], as might the prevention of multimorbidity, which occurs some 10–15 years earlier in socioeconomically disadvantaged groups [49]. Routine assessment of functional ability in primary care could enable earlier identification of disability, when recovery is more achievable.

### Strengths and limitations

Our model uses longitudinal data from three cohort studies, providing data on a large sample of people aged over 50. Combining the three studies also provides strong coverage of age groups from 50 to 54 to over 85, which is important as those aged 85 and over are under-represented in research despite being the fastest growing UK age group [54]. Our statistical method, multistate models, captures transitions to both more and less disabled states, thus allowing for more realistic representations of the underlying disability processes than simpler time-to-event models, such as the Cox proportional hazard models. Lastly, the total life expectancies forecast by our model are comparable to those forecast by the Office for National Statistics.

However, our work has limitations. Firstly, our model compares people living in the most deprived areas to the rest of the population. This was a necessity to simplify the number of parameters in the model, allowing convergence at the parameters of best fit. However, this simplification does preclude differences between the four less deprived quintiles. Additionally, multistate models use the Markov assumption, which asserts that the probability of any transition is only dependent on the currently occupied state and not the history of states previously occupied. Therefore, while the Markov assumption simplifies the modelling process and facilitates computational tractability, it may not capture some dependencies between states and transitions.

We used the English Index of Multiple Deprivation as our measure of socio-economic position, as it is a common metric to all three studies. However, IMD is an area-level rather than an individual-level measure, and substantial within-area heterogeneity may attenuate observed inequalities. As a result, the potential gains in DFLE from reducing socio-economic disadvantage may be underestimated. Individual-level measures such as wealth may more accurately determine the DFLE gap between ‘the richest and poorest’ [55, 56].

A further limitation relates to the assumption that age-associated effects operate proportionally across the life course in the counterfactual scenarios. In reality, interventions may have age-specific impacts or differential effects by deprivation group. Our finding that age-associated interventions widen inequalities applies under the simplifying assumption of uniform proportional change, and alternative assumptions may yield different results. The findings illustrate the scale and combination of changes required under the modelled assumptions, rather than ruling out other potential policy approaches.

In addition, the three cohorts used differ in sampling design, geographic coverage and age structure. Pooled estimates should be interpreted as reflecting transition processes observed within the subgroups of these combined cohorts (e.g. males in deprived areas, males in less deprived areas, females in deprived areas and females in less deprived areas) rather than as precise nationally representative projections.

While the pooled data provide strong coverage of older ages, the 85 + population is drawn disproportionately from the Newcastle 85 + Study, a more deprived regional setting. Although absolute DFLE estimates at these ages may not fully generalise to the English 85 + population, our primary comparisons focus on relative differences between the most deprived quintile and others, which remain interpretable within the pooled sample.

Finally, while we have simulated a range of scenarios, it is possible that alternative intervention designs could produce different distributional or aggregate effects, which extend DFLE and narrow socioeconomic inequalities.

## Conclusions

Increasing DFLE among people aged over 50 by the equivalent of 5 years at birth and narrowing DFLE inequalities is possible with at least: (i) a 30% decrease in the age-associated risk of disability, (ii) a 30% increase in the age-associated risk of recovery from disability, and (iii) a reduction in deprivation-associated risks of disability. Taken on their own, age-based interventions, as modelled here, increase inequalities, as they disproportionately benefit people living in less deprived areas.

## Supplementary Information

Below is the link to the electronic supplementary material.

Supplementary Material 1: Additional file 1: S1 – S12

## Data Availability

ELSA data are available from the UK Data Service ( [http://doi.org/10.5255/UKDA-Series-200011](http:/doi.org/10.5255/UKDA-Series-200011) ). CFAS II data are available from Dementias Platform UK ( [https://doi.org/10.48532/013000](https:/doi.org/10.48532/013000) ). Requests for Newcastle 85+ data can be made via the study website ( [https://research.ncl.ac.uk/85plus/](https:/research.ncl.ac.uk/85plus) ).

## Abbreviations

ADL: Activity of Daily Living
CFAS: Cognitive Function and Ageing Study
DFLE: Disability-Free Life Expectancy
ELSA: English Longitudinal Study of Ageing
IADL: Instrumental Activity of Daily Living
IMaCh: Interpolated Markov Chain (software name)
IMD: English Index of Multiple Deprivation
NIHR: National Institute for Health and Care Research
UK: United Kingdom
