# Supplementary material for: Disability free life expectancy: a simulation study of scenarios to extend healthy years and reduce inequalities
Source: BMC Med. 2026 May 21;24:440. doi: 10.1186/s12916-026-04934-5 (PMC13471628; doi:10.1186/s12916-026-04934-5)
Supplement: Supplementary file 1 [file 12916_2026_4934_MOESM1_ESM.docx]

**Additional file 1**

**Disability Free Life Expectancy:**

**A simulation study of intervention scenarios to extend healthy years and reduce inequalities**

Table of Contents

[Additional file 1: S1 Cohort studies	2](#_Toc229415611)

[Additional file 1: S2 Definition of ADL and IADL/Mobility disability	3](#_Toc229415612)

[Additional file 1: S3 Highlighted four intervention scenarios	5](#_Toc229415613)

[Additional file 1: S4 Deprivation-associated interventions	6](#_Toc229415614)

[Additional file 1: S5 Age-associated interventions	9](#_Toc229415615)

[Additional file 1: S6 Age and Deprivation-associated interventions	12](#_Toc229415616)

[Additional file 1: S7 Transition probabilities	14](#_Toc229415617)

[Additional file 1: S8 Required equivalent increase in Disability Free Life Expectancy	16](#_Toc229415618)

[Additional file 1: S9 Number and weighted percentage of participants in the three contributory datasets	18](#_Toc229415619)

[Additional file 1: S10 Counts of each transition observed in the data.	19](#_Toc229415620)

[Additional file 1: S11 Total life expectancies	20](#_Toc229415621)

[Additional file 1: S12 Baseline model parameters	21](#_Toc229415622)

# Additional file 1: S1 Cohort studies

The English Longitudinal Study of Ageing (ELSA) is a representative panel study of adults aged 50+ in England. It has collected data approximately every two years since 2002. We used data from waves 1-6 (2002-2013), as mortality data is not disclosed beyond wave 6 [45]. ELSA had 11 234 participants in wave 1, with refreshment cohorts added periodically.

The Cognitive Function and Ageing Study II (CFAS II) is a population-based study of adults aged 65+ recruited from three centres in England: Cambridgeshire, Nottingham and Newcastle upon Tyne. Participants were interviewed over two waves (wave 1: 2008-11, wave 2: 2010-13), with 7 762 participants at baseline [46].

The Newcastle 85+ Study is the largest population-based longitudinal study of people aged 85 and older in the world. It recruited 849 people in Newcastle upon Tyne and neighbouring North Tyneside over five waves from age 85 (2006) to age 95 (2016).

Mortality was ascertained through linkage to national death registration data, supplemented by information collected during follow-up interviews where applicable. The CFAS II and Newcastle 85+ studies provide the month and year of birth and death. However, ELSA only provides a year for these dates. We assumed births and deaths occurred at the midpoint of the year, in June.

# Additional file 1: S2 Definition of ADL and IADL/Mobility disability

| **Additional file 1: Table S2** ADL, IADL and Mobility variables present across ELSA, CFAS II and the Newcastle 85+ study | | | |
| --- | --- | --- | --- |
|  | **ELSA** | **CFAS II** | **Newcastle 85+** |
| ADL | Difficulty getting in or out of bed | Bedfast | Are you able to get in and out of bed? |
|  | - | Chairfast | Are you able to get in and out of a chair? |
|  | Difficulty using the toilet, including getting up or down | Are you able to get to and use the toilet? | Are you able to get on and off the toilet? |
|  | Difficulty dressing, including putting on shoes and socks | Are you able to put on your shoes and socks or stockings? | Are you able to dress and undress yourself? |
|  | Difficulty bathing or showering | Are you able to wash all over or bath? | Are you able to wash yourself all over? |
|  | Difficulty eating, such as cutting up food | - | Are you able to feed yourself (including cutting up food)? |
| IADL | Difficulty preparing a hot meal | Are you able to prepare and cook a hot meal? | Are you able to prepare and cook a hot meal? |
|  | Difficulty shopping for groceries | Are you able to shop and carry heavy bags? | Are you able to do your shopping for groceries (including getting your shopping home)? |
|  | Difficulty doing work around the house or garden | Are you able to do the light housework? (e.g. vacuuming, mopping floors, ironing, making beds) | Are you able to do light housework (e.g. dusting and tidying up)? |
|  | - | Are you able to do heavy housework (e.g. cleaning windows, scrubbing floors) | Are you able to do heavy housework (e.g. mopping, vacuuming)? |
|  | Difficulty managing money, such as paying bills and keeping track of expenses | Do you have any difficulty managing money (paying bills/ writing cheques or using an ATM to remove or deposit money)? | Are you able to manage money, such as paying bills and keeping track of expenses? |
|  | Difficulty taking medications | Do you have any difficulty taking medicine (preparing and taking the correct dose)? | Are you able to take your medication? |
| Mobility | Difficulty climbing one or several flights of stairs without resting | Are you able to go up and down stairs? | Are you able to go up and down stairs/steps? |
|  | Difficulty walking across a room | - | Are you able to get around the house? |
|  | Difficulty walking 1/4 mile unaided | No difficulty is usually ambulant non-housebound. | Are you able to walk 400 yards |

ADL: Activity of Daily Living, IADL: Instrumental Activity of Daily Living

ELSA, CFAS II and the Newcastle 85+ Study include slightly different items relating to limitations in ADLs, IADLs, and mobility. For example, ELSA does not distinguish between light and heavy housework, instead asking about ‘work around the house or garden’. The specific items included in each study are detailed in Additional file 1: Table S2.

Question wording and response options also differ between studies. We harmonised measures across cohorts by defining disability states based on the presence of any reported difficulty within the relevant domain (ADL or IADL/mobility), as described below.

In ELSA, participants are asked:

‘Because of a health problem, do you have difficulty doing any of the activities on this card? Exclude any difficulties that you expect to last less than three months’. Responses are binary (mentioned/not mentioned). A report of difficulty with any ADL item was classified as ADL disability, and a report of difficulty with any IADL or mobility item was classified as IADL/mobility disability.

In CFAS II, participants are asked questions of the form: ‘Do you have any difficulty…?’ Response options include ‘no difficulty’, ‘some difficulty’, and ‘needs help’, with ‘some difficulty’ also recorded where an aid is used. Both ‘some difficulty’ and ‘needs help’ were classified as indicating disability within the relevant ADL or IADL/Mobility domain. Some difficulties were assessed through interviewer-rated categories to the question ‘Establish degree of mobility of subject’. These included ‘bedfast’, ‘chairfast’, and ‘usually ambulant non-housebound’.

In the Newcastle 85+ Study, participants are asked questions of the form: ‘Are you able to…?’ Response options include: ‘no difficulty doing this by myself’, ‘some difficulty doing this by myself’, ‘can only do this by myself if I use an aid or appliance’, ‘unable to do this by myself’, and ‘need someone else’s help’. Responses indicating difficulty, use of an aid, inability, or need for help were classified as disability within the relevant ADL or IADL/Mobility domain.

# Additional file 1: S3 Highlighted four intervention scenarios

| Additional file 1: Table S3. Disability Free Life Expectancies in each intervention scenario and the estimated gain from baseline (i.e. no intervention). | | | | | | |
| --- | --- | --- | --- | --- | --- | --- |
| **Scenario** | **Gender** | **Deprivation** | **DFLE** | **CI** | **DLFE gain** | **CI** |
| Baseline | Female | High deprivation | 13.0 | (12.2–13.8) | - | - |
|  |  | Others | 17.2 | (16.8–17.6) | - | - |
|  | Male | High deprivation | 15.1 | (14.1–16.1) | - | - |
|  |  | Others | 20.4 | (20.0–20.9) | - | - |
| 1→2 Deprivation x0.9 | Female | High deprivation | 13.6 | (12.8–14.5) | 0.6 | (-0.6–1.8) |
|  |  | Others | 17.2 | (16.8–17.6) | 0.0 | (0.0–0.0) |
|  | Male | High deprivation | 15.7 | (14.6–16.7) | 0.5 | (-0.9–2.0) |
|  |  | Others | 20.4 | (20.0–20.9) | 0.0 | (0.0–0.0) |
| 1→2,2→3, 2→1, 3→2 Deprivation equity | Female | High deprivation | 15.8 | (14.9–16.7) | 2.8 | (1.6–4.0) |
|  |  | Others | 17.2 | (16.8–17.6) | 0.0 | (0.0–0.0) |
|  | Male | High deprivation | 17.4 | (16.4–18.5) | 2.3 | (0.8–3.8) |
|  |  | Others | 20.4 | (20.0–20.9) | 0.0 | (0.0–0.0) |
| 1→2,2→3 Age x0.60, 2→1,3→2 Age x1.4 | Female | High deprivation | 21.0 | (19.9–22.1) | 8.0 | (6.6–9.3) |
|  |  | Others | 25.9 | (25.3–26.5) | 8.7 | (7.9–9.4) |
|  | Male | High deprivation | 21.4 | (20.1–22.8) | 6.3 | (4.7–8.0) |
|  |  | Others | 26.9 | (26.3–27.5) | 6.5 | (5.7–7.3) |
| 1→2,2→3 Age x0.70, 2→1,3→2 Age x1.3, Deprivation equity | Female | High deprivation | 21.6 | (20.6–22.7) | 8.6 | (7.3–10.0) |
|  |  | Others | 23.5 | (23.0–24.0) | 6.3 | (5.6–6.9) |
|  | Male | High deprivation | 21.9 | (20.6–23.2) | 6.8 | (5.1–8.4) |
|  |  | Others | 25.2 | (24.7–25.8) | 4.8 | (4.1–5.5) |
| DFLE – Disability Free Life Expectancy; CI – 95% Confidence Interval | | | | | | |

IMaCh provides standard errors for DFLE estimates derived from the multinomial logistic transition models using maximum likelihood estimation. We report 95% confidence intervals for absolute DFLE estimates under each intervention scenario in Additional file 1: Table S3. To estimate uncertainty around gains in DFLE (difference between intervention and baseline), we propagated uncertainty assuming independence between estimates, as the full covariance structure between baseline and counterfactual scenarios is not available. This approach is likely to produce conservative (overestimated) confidence intervals for the gain estimates.

# Additional file 1: S4 Deprivation-associated interventions

*Additional file 1: Figure S4. Changes in DFLE for interventions which narrow the gap between the most deprived population quintile and the rest of the population. As these interventions narrow the deprivation gap, they only increase DFLE for the most deprived quintile. Numbers in the dots indicate the increase in DFLE associated with each intervention. Interventions are listed on the y-axis. The format x ⟶ y represents the state transition(s) the intervention corresponds to: (1) Independent, (2) IADL/mobility disability, (3) ADL disability and (4) Death. The multiplication factor shows the change in the probability of the transition, with the probability of transitions to more disabled states decreasing and less disabled states increasing. Equity indicates that the probability of a transition for people on the most deprived quintile is the same as those in the other four quintiles.*

| **Additional file 1: Table S4** Disability Free Life Expectancies in each deprivation-based intervention scenario and the estimated gain from baseline (i.e. no intervention). | | | | | | |
| --- | --- | --- | --- | --- | --- | --- |
| **Scenario** | **Gender** | **Deprivation** | **DFLE** | **CI** | **DLFE gain** | **CI** |
| 1→2,2→3, 2→1, 3→2 Deprivation equity | Female | High deprivation | 15.8 | (14.9–16.7) | 2.8 | (1.6–4.0) |
|  |  | Others | 17.2 | (16.8–17.6) | 0.0 | (0.0–0.0) |
|  | Male | High deprivation | 17.4 | (16.4–18.5) | 2.3 | (0.8–3.8) |
|  |  | Others | 20.4 | (20.0–20.9) | 0.0 | (0.0–0.0) |
| 3→2 Deprivation equity | Female | High deprivation | 13.0 | (12.2–13.9) | 0.0 | (-1.2–1.2) |
|  |  | Others | 17.2 | (16.8–17.6) | 0.0 | (0.0-0.0) |
|  | Male | High deprivation | 15.2 | (14.2–16.1) | 0.0 | (-1.4–1.4) |
|  |  | Others | 20.4 | (20.0–20.9) | 0.0 | (0.0-0.0) |
| 2→3 Deprivation equity | Female | High deprivation | 13.3 | (12.5–14.1) | 0.3 | (-0.9–1.5) |
|  |  | Others | 17.2 | (16.8–17.6) | 0.0 | (0.0-0.0) |
|  | Male | High deprivation | 15.4 | (14.4–16.4) | 0.3 | (-1.1–1.7) |
|  |  | Others | 20.4 | (20.0–20.9) | 0.0 | (0.0-0.0) |
| 2→3 Deprivation x0.9 | Female | High deprivation | 13.2 | (12.3–14.0) | 0.2 | (-1.0–1.3) |
|  |  | Others | 17.2 | (16.8–17.6) | 0.0 | (0.0-0.0) |
|  | Male | High deprivation | 15.3 | (14.3–16.3) | 0.1 | (-1.3–1.5) |
|  |  | Others | 20.4 | (20.0–20.9) | 0.0 | (0.0-0.0) |
| 2→1 Deprivation equity | Female | High deprivation | 14.4 | (13.6–15.3) | 1.4 | (0.2–2.6) |
|  |  | Others | 17.2 | (16.8–17.6) | 0.0 | (0.0-0.0) |
|  | Male | High deprivation | 16.3 | (15.3–17.3) | 1.2 | (-0.2–2.6) |
|  |  | Others | 20.4 | (20.0–20.9) | 0.0 | (0.0-0.0) |
| 2→1 Deprivation x1.2 | Female | High deprivation | 13.9 | (13.0–14.7) | 0.8 | (-0.3–2.0) |
|  |  | Others | 17.2 | (16.8–17.6) | 0.0 | (0.0-0.0) |
|  | Male | High deprivation | 15.8 | (14.8–16.8) | 0.7 | (-0.7–2.1) |
|  |  | Others | 20.4 | (20.0–20.9) | 0.0 | (0.0-0.0) |
| 2→1 Deprivation x1.1 | Female | High deprivation | 13.4 | (12.6–14.3) | 0.4 | (-0.7–1.6) |
|  |  | Others | 17.2 | (16.8–17.6) | 0.0 | (0.0-0.0) |
|  | Male | High deprivation | 15.5 | (14.5–16.5) | 0.4 | (-1.0–1.8) |
|  |  | Others | 20.4 | (20.0–20.9) | 0.0 | (0.0-0.0) |
| 1→4 Deprivation equity | Female | High deprivation | 13.0 | (12.2–13.8) | 0.0 | (-1.2–1.2) |
|  |  | Others | 17.2 | (16.8–17.6) | 0.0 | (0.0-0.0) |
|  | Male | High deprivation | 15.1 | (14.1–16.1) | 0.0 | (-1.4–1.4) |
|  |  | Others | 20.4 | (20.0–20.9) | 0.0 | (0.0-0.0) |
| 1→4 Deprivation x0.6 | Female | High deprivation | 13.1 | (12.3–14.0) | 0.1 | (-1.1–1.3) |
|  |  | Others | 17.2 | (16.8–17.6) | 0.0 | (0.0-0.0) |
|  | Male | High deprivation | 15.4 | (14.4-16.5) | 0.3 | (-1.1–1.7) |
|  |  | Others | 20.4 | (20.0–20.9) | 0.0 | (0.0-0.0) |
| 1→4 Deprivation x0.7 | Female | High deprivation | 13.1 | (12.3–13.9) | 0.1 | (-1.1–1.3) |
|  |  | Others | 17.2 | (16.8–17.6) | 0.0 | (0.0-0.0) |
|  | Male | High deprivation | 15.4 | (14.3–16.4) | 0.2 | (-1.2–1.6) |
|  |  | Others | 20.4 | (20.0–20.9) | 0.0 | (0.0-0.0) |
| 1→4 Deprivation x0.8 | Female | High deprivation | 13.1 | (12.2–13.9) | 0.1 | (-1.1–1.2) |
|  |  | Others | 17.2 | (16.8–17.6) | 0.0 | (0.0-0.0) |
|  | Male | High deprivation | 15.3 | (14.3–16.3) | 0.1 | (-1.3–1.6) |
|  |  | Others | 20.4 | (20.0–20.9) | 0.0 | (0.0-0.0) |
| 1→4 Deprivation x0.9 | Female | High deprivation | 13.0 | (12.2–13.9) | 0.0 | (-1.2–1.2) |
|  |  | Others | 17.2 | (16.8–17.6) | 0.0 | (0.0-0.0) |
|  | Male | High deprivation | 15.2 | (14.2–16.2) | 0.1 | (-1.3–1.5) |
|  |  | Others | 20.4 | (20.0–20.9) | 0.0 | (0.0-0.0) |
| 1→3 Deprivation equity | Female | High deprivation | 13.4 | (12.6–14.2) | 0.4 | (-0.8–1.6) |
|  |  | Others | 17.2 | (16.8–17.6) | 0.0 | (0.0-0.0) |
|  | Male | High deprivation | 15.8 | (14.9–16.7) | 0.7 | (-0.7–2.0) |
|  |  | Others | 20.4 | (20.0–20.9) | 0.0 | (0.0-0.0) |
| 1→3 Deprivation x0.8 | Female | High deprivation | 13.3 | (12.5–14.2) | 0.3 | (-0.8–1.5) |
|  |  | Others | 17.2 | (16.8–17.6) | 0.0 | (0.0-0.0) |
|  | Male | High deprivation | 15.7 | (14.8–16.6) | 0.6 | (-0.8–1.9) |
|  |  | Others | 20.4 | (20.0–20.9) | 0.0 | (0.0-0.0) |
| 1→3 Deprivation x0.9 | Female | High deprivation | 13.2 | (12.3–14.0) | 0.2 | (-1.0–1.3) |
|  |  | Others | 17.2 | (16.8–17.6) | 0.0 | (0.0-0.0) |
|  | Male | High deprivation | 15.4 | (14.5–16.4) | 0.3 | (-1.1–1.7) |
|  |  | Others | 20.4 | (20.0–20.9) | 0.0 | (0.0-0.0) |
| 1→2 Deprivation equity | Female | High deprivation | 14.1 | (13.2–14.9) | 1.0 | (-0.2–2.2) |
|  |  | Others | 17.2 | (16.8–17.6) | 0.0 | (0.0-0.0) |
|  | Male | High deprivation | 16.0 | (14.9–17.1) | 0.9 | (-0.6–2.3) |
|  |  | Others | 20.4 | (20.0–20.9) | 0.0 | (0.0-0.0) |
| 1→2 Deprivation x0.9 | Female | High deprivation | 13.6 | (12.8–14.5) | 0.6 | (-0.6–1.8) |
|  |  | Others | 17.2 | (16.8–17.6) | 0.0 | (0.0-0.0) |
|  | Male | High deprivation | 15.7 | (14.6–16.7) | 0.5 | (-0.9–2.0) |
|  |  | Others | 20.4 | (20.0–20.9) | 0.0 | (0.0-0.0) |
| DFLE – Disability Free Life Expectancy; CI – 95% Confidence Interval | | | | | | |

IMaCh provides standard errors for DFLE estimates derived from the multinomial logistic transition models using maximum likelihood estimation. We report 95% confidence intervals for absolute DFLE estimates under each intervention scenario in Additional file 1: Figure S4 and Table S4. To estimate uncertainty around gains in DFLE (difference between intervention and baseline), we propagated uncertainty assuming independence between estimates, as the full covariance structure between baseline and counterfactual scenarios is not available. This approach is likely to produce conservative (overestimated) confidence intervals for the gain estimates.

# Additional file 1: S5 Age-associated interventions

*Additional file 1: Figure S5 Changes in DFLE for interventions which reduce the association with ageing. Numbers in the dots indicate the increase in DFLE associated with each intervention. Interventions are listed on the y-axis. The format x ⟶ y represents the state transition(s) the intervention corresponds to: (1) Independent, (2) IADL/mobility disability, (3) ADL disability and (4) Death. The multiplication factor shows the change in the probability of the transition, with the probability of transitions to more disabled states decreasing and less disabled states increasing.*

| **Additional file 1: Table S5** Disability Free Life Expectancies in each age-based intervention scenario and the estimated gain from baseline (i.e. no intervention). | | | | | | |
| --- | --- | --- | --- | --- | --- | --- |
| **Scenario** | **Gender** | **Deprivation** | **DFLE** | **CI** | **DLFE gain** | **CI** |
| 1→2,2→3 Age x0.50, 2→1,3→2 Age x1.5 | Female | High deprivation | 23.3 | (22.1–24.6) | 10.3 | (8.8–11.8) |
|  |  | Others | 28.4 | (27.7–29.2) | 11.2 | (10.4–12.1) |
|  | Male | High deprivation | 23.1 | (21.6–24.5) | 7.9 | (6.1–9.7) |
|  |  | Others | 28.6 | (27.9–29.3) | 8.2 | (7.3–9.0) |
| 1→2,2→3 Age x0.60, 2→1,3→2 Age x1.4 | Female | High deprivation | 21.0 | (19.9–22.1) | 8.0 | (6.6–9.3) |
|  |  | Others | 25.9 | (25.3–26.5) | 8.7 | (7.9–9.4) |
|  | Male | High deprivation | 21.4 | (20.1–22.8) | 6.3 | (4.7–8.0) |
|  |  | Others | 26.9 | (26.3–27.5) | 6.5 | (5.7–7.3) |
| 1→2,2→3 Age x0.70, 2→1,3→2 Age x1.3 | Female | High deprivation | 18.7 | (17.7–19.7) | 5.7 | (4.4–7.0) |
|  |  | Others | 23.5 | (23.0–24.0) | 6.3 | (5.6–6.9) |
|  | Male | High deprivation | 19.8 | (18.6–21.0) | 4.7 | (3.1–6.2) |
|  |  | Others | 25.2 | (24.7–25.8) | 4.8 | (4.1–5.5) |
| 1→2,2→3 Age x0.80, 2→1,3→2 Age x1.2 | Female | High deprivation | 16.7 | (15.7–17.6) | 3.7 | (2.4–4.9) |
|  |  | Others | 21.2 | (20.7–21.7) | 4.0 | (3.4–4.6) |
|  | Male | High deprivation | 18.2 | (17.1–19.3) | 3.1 | (1.6–4.6) |
|  |  | Others | 23.6 | (23.1–24.1) | 3.2 | (2.5–3.8) |
| 1→2,2→3 Age x0.90, 2→1,3→2 Age x1.1 | Female | High deprivation | 14.8 | (13.9–15.6) | 1.8 | (0.5–3.0) |
|  |  | Others | 19.1 | (18.7–19.6) | 1.9 | (1.3–2.5) |
|  | Male | High deprivation | 16.6 | (15.6–17.7) | 1.5 | (0.1–2.9) |
|  |  | Others | 22.0 | (21.5–22.4) | 1.5 | (0.9–2.2) |
| 1→2,2→3 Age x0.95, 2→1,3→2 Age x1.05 | Female | High deprivation | 13.9 | (13.0–14.7) | 0.9 | (-0.3–2.1) |
|  |  | Others | 18.1 | (17.7–18.6) | 0.9 | (0.3–1.6) |
|  | Male | High deprivation | 15.9 | (14.9–16.9) | 0.7 | (-0.7–2.2) |
|  |  | Others | 21.2 | (20.7–21.6) | 0.8 | (0.1–1.4) |
| 3→2 Age x1.1 | Female | High deprivation | 13.2 | (12.3–14.0) | 0.2 | (-1.0–1.3) |
|  |  | Others | 17.3 | (16.9–17.8) | 0.1 | (-0.5–0.7) |
|  | Male | High deprivation | 15.3 | (14.3–16.3) | 0.2 | (-1.2–1.6) |
|  |  | Others | 20.5 | (20.1–21.0) | 0.1 | (-0.6–0.7) |
| 3→2 Age x1.05 | Female | High deprivation | 13.1 | (12.3–13.9) | 0.1 | (-1.1–1.3) |
|  |  | Others | 17.3 | (16.8–17.7) | 0.1 | (-0.5–0.7) |
|  | Male | High deprivation | 15.2 | (14.2–16.2) | 0.1 | (-1.3–1.5) |
|  |  | Others | 20.5 | (20.0–20.9) | 0.0 | (-0.6–0.7) |
| 2→3 Age x0.90 | Female | High deprivation | 13.2 | (12.4–14.0) | 0.2 | (-1.0–1.4) |
|  |  | Others | 17.4 | (17.0–17.8) | 0.2 | (-0.4–0.8) |
|  | Male | High deprivation | 15.3 | (14.3–16.3) | 0.2 | (-1.2–1.6) |
|  |  | Others | 20.6 | (20.1–21.0) | 0.1 | (-0.5–0.8) |
| 2→3 Age x0.95 | Female | High deprivation | 13.1 | (12.3–13.9) | 0.1 | (-1.1–1.3) |
|  |  | Others | 17.3 | (16.9–17.7) | 0.1 | (-0.5–0.7) |
|  | Male | High deprivation | 15.2 | (14.2–16.2) | 0.1 | (-1.3–1.5) |
|  |  | Others | 20.5 | (20.0–20.9) | 0.1 | (-0.6–0.7) |
| 2→1 Age x1.1 | Female | High deprivation | 13.6 | (12.7–14.4) | 0.5 | (-0.6–1.7) |
|  |  | Others | 17.8 | (17.4–18.2) | 0.6 | (0.0–1.2) |
|  | Male | High deprivation | 15.6 | (14.6–16.6) | 0.5 | (-0.9–1.9) |
|  |  | Others | 20.9 | (20.5–21.4) | 0.5 | (-0.2–1.1) |
| 2→1 Age x1.05 | Female | High deprivation | 13.3 | (12.4–14.1) | 0.3 | (-0.9–1.5) |
|  |  | Others | 17.5 | (17.1–17.9) | 0.3 | (-0.3–0.9) |
|  | Male | High deprivation | 15.4 | (14.4–16.4) | 0.2 | (-1.2–1.6) |
|  |  | Others | 20.7 | (20.2–21.1) | 0.2 | (-0.4–0.9) |
| 1→2 Age x0.95 | Female | High deprivation | 13.4 | (12.6–14.2) | 0.4 | (-0.8–1.6) |
|  |  | Others | 17.7 | (17.2–18.1) | 0.5 | (-0.2–1.1) |
|  | Male | High deprivation | 15.5 | (14.4–16.5) | 0.3 | (-1.1–1.8) |
|  |  | Others | 20.8 | (20.4–21.3) | 0.4 | (-0.2–1.0) |
| DFLE – Disability Free Life Expectancy; CI – 95% Confidence Interval | | | | | | |

IMaCh provides standard errors for DFLE estimates derived from the multinomial logistic transition models using maximum likelihood estimation. We report 95% confidence intervals for absolute DFLE estimates under each intervention scenario in Additional file 1: Figure S5 and Table S5. To estimate uncertainty around gains in DFLE (difference between intervention and baseline), we propagated uncertainty assuming independence between estimates, as the full covariance structure between baseline and counterfactual scenarios is not available. This approach is likely to produce conservative (overestimated) confidence intervals for the gain estimates.

# Additional file 1: S6 Age and Deprivation-associated interventions

*Additional file 1: Figure S6 Changes in DFLE for interventions which narrow the gap between the most deprived population quintile and the rest of the population and reduce the association with ageing. Numbers in the dots indicate the increase in DFLE associated with each intervention. Interventions are listed on the y-axis. The format x ⟶ y represents the state transition(s) the intervention corresponds to: (1) Independent, (2) IADL/mobility disability, (3) ADL disability and (4) Death. The multiplication factor shows the change in the probability of the transition, with the probability of transitions to more disabled states decreasing and less disabled states increasing. Equity indicates that the probability of a transition for people in the most deprived quintile is the same as those in the other four quintiles.*

| **Additional file 1: Table S6** Disability Free Life Expectancies in combined age and deprivation-based intervention scenarios and the estimated gain from baseline (i.e. no intervention). | | | | | | |
| --- | --- | --- | --- | --- | --- | --- |
| **Scenario** | **Gender** | **Deprivation** | **DFLE** | **CI** | **DLFE gain** | **CI** |
| 1→2,2→3 Age x0.60, 2→1,3→2 Age x1.4, Deprivation equity | Female | High deprivation | 25.9 | (24.5–27.3) | 12.9 | (11.3–14.5) |
|  |  | Others | 28.4 | (27.7–29.2) | 11.2 | (10.4–12.1) |
|  | Male | High deprivation | 24.7 | (23.2–26.3) | 9.6 | (7.7–11.5) |
|  |  | Others | 28.6 | (27.9–29.3) | 8.2 | (7.3–9.0) |
| 1→2,2→3 Age x0.70, 2→1,3→2 Age x1.3, Deprivation equity | Female | High deprivation | 21.6 | (20.6–22.7) | 8.6 | (7.3–10.0) |
|  |  | Others | 23.5 | (23.0–24.0) | 6.3 | (5.6–6.9) |
|  | Male | High deprivation | 21.9 | (20.6–23.2) | 6.8 | (5.1–8.4) |
|  |  | Others | 25.2 | (24.7–25.8) | 4.8 | (4.1–5.5) |
| 1→2,2→3 Age x0.80, 2→1,3→2 Age x1.2, Deprivation equity | Female | High deprivation | 19.6 | (18.6–20.6) | 6.6 | (5.3–7.9) |
|  |  | Others | 21.2 | (20.7–21.7) | 4.0 | (3.4–4.6) |
|  | Male | High deprivation | 20.4 | (19.2–21.6) | 5.3 | (3.7–6.9) |
|  |  | Others | 23.6 | (23.1–24.1) | 3.2 | (2.5–3.8) |
| 1→2,2→3 Age, Deprivation x0.90, 2→1,3→2 Age, Deprivation x1.1 | Female | High deprivation | 15.8 | (14.9–16.7) | 2.8 | (1.6–4.0) |
|  |  | Others | 18.8 | (18.4–19.2) | 1.6 | (1.0–2.2) |
|  | Male | High deprivation | 17.4 | (16.3–18.5) | 2.3 | (0.8–3.8) |
|  |  | Others | 21.8 | (21.3–22.2) | 1.3 | (0.7–2.0) |
| DFLE – Disability Free Life Expectancy; CI – 95% Confidence Interval | | | | | | |

IMaCh provides standard errors for DFLE estimates derived from the multinomial logistic transition models using maximum likelihood estimation. We report 95% confidence intervals for absolute DFLE estimates under each intervention scenario in Additional file 1: Figure S6 and Table 6. To estimate uncertainty around gains in DFLE (difference between intervention and baseline), we propagated uncertainty assuming independence between estimates, as the full covariance structure between baseline and counterfactual scenarios is not available. This approach is likely to produce conservative (overestimated) confidence intervals for the gain estimates.

# Additional file 1: S7 Transition probabilities

The multistate model was modelled in IMaCh (euroreves.ined.fr/imach). IMaCh calculates transition probabilities using a multinomial logistic model.

In our model, the probability of moving from state *i* to state *j,* $p_{ij}$, relative to the probability of remaining in state *i*, $p_{ii}$, is calculated by:

$\ln\left( \frac{p_{ij}}{p_{ii}} \right)=a_{ij}+b_{ij}\cdot age+c_{ij}\cdot sex+d_{ij}\cdot\mathrm{deprivation}$ ,

(1)

where $a_{ij}, b_{ij}, c_{ij} \mathrm{and} d_{ij}$ are constants and ${p_{ij}}/{p_{ii}}$ is the hazard ratio. Sex is a variable with values 0 for women and 1 for men. Deprivation is a variable with values 1 for people living in the most deprived areas (quintile 1) and 0 for the rest of the population. Age is a continuous variable measured in months.

Changing the values of the constants associated with age, sex and deprivation, $b_{ij}, c_{ij} \mathrm{and} d_{ij}$ can thus increase or decrease the probability of a transition. Introducing a constant, $\alpha$, to modify the probability of a transition, and rearranging equation (1) yields

$d_{ij}^{'} \cdot deprivation = ln \left( \frac{\alpha\cdot p_{ij}}{p_{ii}} \right)- a_{ij}-b_{ij}\cdot age- c_{ij}\cdot\mathrm{sex}$ ,

(2)

which calculates the new value of $d_{ij}^{'} \cdot\mathrm{deprivation}$ to change the probability of the transition occurring by a factor of $\alpha$. This allows us to model an intervention which changes the probability of transitions from being in the most deprived group, for example. As $\mathrm{deprivation}=1$ for the most deprived group, equation (2) simplifies to

$d_{ij}^{'} = ln \left( \frac{p_{ij}^{'}}{p_{ii}} \right)- a_{ij}-b_{ij}\cdot age- c_{ij}\cdot\mathrm{sex}$ ,

(3)

where $p_{ij}^{'}=\alpha\cdot p_{ij} .$

Similar approaches can be used to change the probability of transitions by modifying the parameters associated with the other covariates.

# Additional file 1: S8 Required equivalent increase in Disability Free Life Expectancy

To meet the previous government targets in place when this study was commissioned [20], DFLE needs to increase by 5 years for newborns. As the multistate model in this study is parameterised from age 50 onwards, we derived an equivalent benchmark for adults aged 50. This ‘target equivalence’ step was used as a calibration procedure to translate a policy target defined at birth into a comparable quantitative objective within the modelling framework. Here we calculate the equivalent increase for adults aged 50.

The Office for National Statistics publishes DFLEs by deprivation decile [47]. These calculations are cross-sectional. To convert the decile estimates to estimates for the most deprived quintile, the median of the DFLE estimates for deciles 1 and 2 was used. The median of the estimates for deciles 3-10 was used for the rest of the population.

The Ageing Grand Challenge was defined in 2018 when the DFLE for the most deprived quintile was 54.3 years for men aged <1 years and 52.7 years for women. Increasing this by 5 years, to 59.3 years and 57.7 years, requires a proportional increase of 9.2% and 9.5%, respectively. We applied proportional scaling because it provides a neutral way to express the magnitude of improvement required while preserving the structure of DFLE differences between groups.

The total DFLE of a man aged 50 in the most deprived quintile was 64.5 years in 2018. Increasing this by 9.2% yields 70.4 years, a difference of 5.9 years. For women, this is 64.2 years increased to 70.3 years, an increase of 6.1 years.

These calculations are provided in Additional file 1: Table S8, along with the equivalent calculation for the rest of the population (deprivation quintiles 2-5).

| Additional file 1: Table S8 DFLE increase at age 50 to be equivalent to +5 years at age 0 | | | | | |
| --- | --- | --- | --- | --- | --- |
| **Deprivation** | **Gender** | **DFLE at age <1** | **Proportional increase for +5 years** | **DFLE at age 50** | **DFLE age 50 x proportional increase** |
| High deprivation | Male | 54.3 | 0.092 | 64.5 | 5.9 |
| High deprivation | Female | 52.7 | 0.095 | 64.2 | 6.1 |
| Others | Male | 64.6 | 0.077 | 71.3 | 5.5 |
| Others | Female | 63.3 | 0.079 | 71.0 | 5.6 |

# Additional file 1: S9 Number and weighted percentage of participants in the three contributory datasets

| Additional file 1: Table S9. Number and percentage of participants in the three contributory datasets, by age, deprivation quintile and gender. ELSA – English Longitudinal Study of Ageing; CFAS II – Cognitive Function and Ageing Study II. Counts are given for the first appearance of each participant with longitudinal data. | | | | | | | | | | | | |
| --- | --- | --- | --- | --- | --- | --- | --- | --- | --- | --- | --- | --- |
|  | ELSA | | | | CFAS II | | | | Newcastle 85+ | | | |
| Characteristic | Male | (%) | Female | (%) | Male | (%) | Female | (%) | Male | (%) | Female | (%) |
| Total | 4683 | (-) | 5631 | (-) | 2704 | (-) | 3048 | (-) | 319 | (-) | 514 | (-) |
| Age |  |  |  |  |  |  |  |  |  |  |  |  |
| 50-54 | 808 | (17.3) | 1006 | (17.9) | - | - | - | - | - | - | - | - |
| 55-59 | 894 | (19.1) | 1027 | (18.2) | - | - | - | - | - | - | - | - |
| 60-64 | 715 | (15.3) | 793 | (14.1) | - | - | - | - | - | - | - | - |
| 65-69 | 733 | (15.7) | 818 | (14.5) | 818 | (30.3) | 811 | (26.6) | - | - | - | - |
| 70-74 | 615 | (13.1) | 723 | (12.8) | 696 | (25.7) | 707 | (23.2) | - | - | - | - |
| 75-79 | 459 | (9.8) | 557 | (9.9) | 560 | (20.7) | 636 | (20.9) | - | - | - | - |
| 80-84 | 289 | (6.2) | 455 | (8.1) | 392 | (14.5) | 512 | (16.8) | 51 | (17.2) | 61 | (11.9) |
| ≥85 | 170 | (3.6) | 252 | (4.5) | 238 | (8.8) | 382 | (12.5) | 268 | (82.8) | 453 | (88.1) |
|  |  |  |  |  |  |  |  |  |  |  |  |  |
| Deprivation |  |  |  |  |  |  |  |  |  |  |  |  |
| 1 (Most) | 683 | (14.6) | 858 | (15.2) | 379 | (14.0) | 475 | (15.6) | 78 | (24.5) | 86 | (16.7) |
| 2 | 860 | (18.4) | 1039 | (18.5) | 471 | (17.4) | 559 | (18.3) | 62 | (19.4) | 112 | (21.8) |
| 3 | 954 | (20.4) | 1160 | (20.6) | 544 | (20.1) | 623 | (20.4) | 60 | (18.8) | 102 | (19.8) |
| 4 | 1098 | (23.4) | 1290 | (22.9) | 634 | (23.4) | 686 | (22.5) | 54 | (16.9) | 111 | (21.6) |
| 5 (Least) | 1088 | (23.2) | 1284 | (22.8) | 676 | (25.0) | 705 | (23.1) | 65 | (20.4) | 103 | (20.0) |

# Additional file 1: S10 Counts of each transition observed in the data.

| Additional file 1: Table S10. Counts of each transition from harmonised survey data. Transition states are 1) Independent, 2) Mobility/IADL, 3) ADL, 4) Death. | |
| --- | --- |
| **Transition** | **Count** |
| 1⟶1 | 16 947 |
| 1⟶2 | 3 413 |
| 1⟶3 | 1 243 |
| 1⟶4 | 406 |
| 2⟶1 | 2 465 |
| 2⟶2 | 6 536 |
| 2⟶3 | 2 747 |
| 2⟶4 | 974 |
| 3⟶1 | 795 |
| 3⟶2 | 2 159 |
| 3⟶3 | 5 897 |
| 3⟶4 | 2 364 |

Participants are observed across multiple survey waves, so each participant may contribute multiple transition observations, as well as being observed to not change state, in Additional file 1: Table S10. State 4 (Death) is an absorbing state, thus there are no transitions away from state 4.

# Additional file 1: S11 Total life expectancies

*Additional file 1: Figure S11. Total life expectancy for men and women forecast by the multistate model used in this study, and the Office for National Statistics (ONS) [35]. This plot uses ONS total life expectancies for 2010-2012, corresponding to the end of data collection for ELSA wave 6 (2012), CFAS II (2011) and Newcastle 85+ (wave 5, 2011/12). As noted by the ONS, life expectancy improvements since 2012 have been slow, and were at similar levels in 2022 (the most recent publication year at the time of writing) and 2012 [35].*

# Additional file 1: S12 Baseline model parameters

| Additional file 1: Table S12 Parameters (and standard errors) of the DFLE model with no interventions. Parameters are input to equation (1) in Supplementary Information S3. Bold parameters are significant at the 95% confidence interval. Transition states are 1) Independent, 2) Mobility/IADL, 3) ADL, and 4) Death. | | | | | | | | |
| --- | --- | --- | --- | --- | --- | --- | --- | --- |
| Transition | Constant | | Age | | Gender: (women, ref: men) | | Deprivation (high deprivation, ref: others) | |
| 1⟶2 | **-8.96** | (0.16) | **0.060** | (0.002) | **0.48** | (0.04) | **0.16** | (0.06) |
| 1⟶3 | **-7.95** | (0.58) | **0.025** | (0.009) | **-0.29** | (0.13) | 0.28 | (0.18) |
| 1⟶4 | **-15.70** | (0.71) | **0.116** | (0.010) | **-0.63** | (0.19) | **0.62** | (0.21) |
| 2⟶1 | **-1.68** | (0.17) | **-0.036** | (0.002) | **-0.14** | (0.05) | **-0.30** | (0.07) |
| 2⟶3 | **-7.02** | (0.18) | **0.041** | (0.002) | **-0.11** | (0.05) | **0.19** | (0.06) |
| 2⟶4 | **-12.58** | (0.42) | **0.092** | (0.005) | **-0.52** | (0.10) | 0.16 | (0.13) |
| 3⟶1 | **-2.06** | (0.44) | **-0.044** | (0.006) | **-0.98** | (0.17) | **-1.94** | (0.54) |
| 3⟶2 | **-3.65** | (0.19) | **-0.008** | (0.003) | **0.18** | (0.06) | -0.01 | (0.06) |
| 3⟶4 | **-11.16** | (0.21) | **0.084** | (0.003) | **-0.45** | (0.05) | 0.09 | (0.06) |
